# Mapping Mind-Brain Development: Towards a Comprehensive Theory

**DOI:** 10.3390/jintelligence8020019

**Published:** 2020-04-26

**Authors:** George Spanoudis, Andreas Demetriou

**Affiliations:** 1 Psychology Department, University of Cyprus, 1678 Nicosia, Cyprus; 2 Department of Psychology, University of Nicosia, 1700 Nicosia, Cyprus; ademetriou@ucy.ac.cy; 3 Cyprus Academy of Science, Letters, and Arts, 1700 Nicosia, Cyprus

**Keywords:** brain, mind, development, intelligence, mental and brain changes, brain networks

## Abstract

The relations between the developing mind and developing brain are explored. We outline a theory of intellectual development postulating that the mind comprises four systems of processes (domain-specific, attention and working memory, reasoning, and cognizance) developing in four cycles (episodic, realistic, rule-based, and principle-based representations, emerging at birth, 2, 6, and 11 years, respectively), with two phases in each. Changes in reasoning relate to processing efficiency in the first phase and working memory in the second phase. Awareness of mental processes is recycled with the changes in each cycle and drives their integration into the representational unit of the next cycle. Brain research shows that each type of processes is served by specialized brain networks. Domain-specific processes are rooted in sensory cortices; working memory processes are mainly rooted in hippocampal, parietal, and prefrontal cortices; abstraction and alignment processes are rooted in parietal, frontal, and prefrontal and medial cortices. Information entering these networks is available to awareness processes. Brain networks change along the four cycles, in precision, connectivity, and brain rhythms. Principles of mind-brain interaction are discussed.

## 1. Introduction

The brain is a biophysical system collecting physical messages from the environment with self as the reference system. It uses this information to construct a representation of the environment to guide action by its bearer. Representations, ensuing understanding, action planning, and the psychological mechanisms involved comprise the mind. Therefore, the brain is the biological substrate of the mind, because the mind emerges, in all of its expressions, from the structure and functioning of the brain. In this paper, we examine how these levels of analyzing mental functions, both the psychological and the biological, relate with each other. The frame for discussion is current research and theory on the organization, functioning, and development of (i) intellectual processes and (ii) the human brain. The paper is organized in two parts: the first focuses on the architecture and functioning and the second on the development of mind and brain. In each of these parts, we first outline the basic findings and principles about the mind and then present evidence and theory about the underlying brain structures and mechanisms. The paper aims to answer the following four questions:

1. Is the architecture of mind suggested by psychological research reflected in the organization and functioning of the brain?
2. What are the neuronal processes implementing mental processes and mechanisms, such as abstraction and reasoning?
3. How are developmental changes in gross parameters of the brain (such as increases in neuronal volume, myelination, networking, and pruning) reflected in developmental changes of cognitive processes?
4. Are there systematic individual differences in brain architecture and functioning that may be connected to individual differences in intellectual functioning and development?

We caution that answering these questions is not an easy matter. On the one hand, psychological research involves: (i) observable responses expressed in different scales and (ii) subjective experiences expressed in various modes, such as self-descriptions, self-evaluations, certainty evaluations, etc. On the other hand, brain research involves biological entities, such as the nature, volume, and organization of neuronal matter and its functional correlates, such as brain rhythms, expressed through various modes (e.g., blood supply and glucose consumption of activated brain areas and electrochemical activity). Structurally, the human brain comprises a large number of cytoarchitecturally discrete regions, such as (Brodmann 1909) areas or more detailed maps identified by recent research (Glasser et al. 2016). Despite the diversity of brain regions, the brain operates as a single integrated entity governed by multiple processes running within a highly constrained and genetically organized context interacting with the environment (Stiles and Jernigan 2010). The scales and precision of measuring each of these dimensions vary both within and across the two levels of description. Thus, we often do not know how to map the different levels of analysis onto each other and when we map them, precision falls short of the optimal. Martínez et al. (2015) recently demonstrated dramatically that the relations between different aspects of cognition, such as fluid intelligence and working memory, cannot be consistently mapped on the same brain dimensions, when the methods of brain mapping vary. With these reservations granted, we will attempt to set a tentative frame for bridging the two fields.

## 2. Architecture

**Figure 1:**
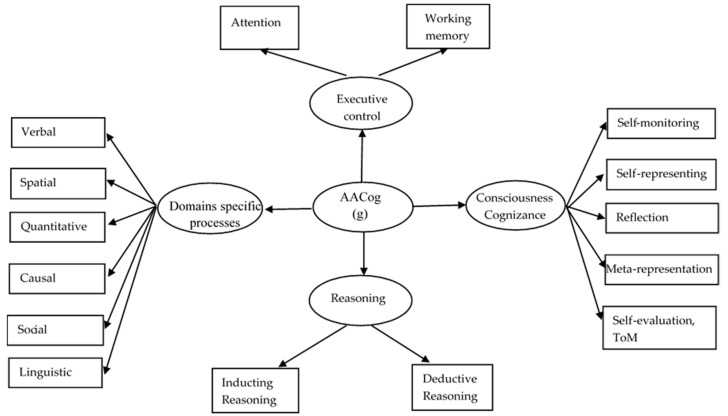
The cognitive architecture of the mind. Note: AACog stands for Abstraction, Alignment, and Cognizance.

The human mind is a hierarchical universe involving multiple systems at different levels of organization. Systems at different levels involve processes serving different tasks during understanding and problem solving. Some are domain-specific, dealing with particular types of information and relations in the environment and some are general, dealing with the relations between mental entities and the production of new ones. Overall, cognitive and psychometric research (Demetriou and Spanoudis 2018) suggests four types of systems: domain-specific, attention and memory, integrative, and cognizance systems. Figure 1 illustrates this architecture through the conventions of structural equation modeling. It can be seen that domain-specific systems are related to a common factor, *g*; in turn, this factor is strongly related with markers of domain-general representational (i.e., attention and working memory), integrative (i.e., reasoning), and self-awareness (i.e., cognizance) processes. This model was tested in several studies presented elsewhere (Demetriou and Spanoudis 2018; Demetriou et al. 2002, 2017, 2018; Makris et al. 2017). Below we focus on each type of system, outlining its psychological profile first and then discussing relevant brain research. To facilitate the reader to map the psychological architecture of the mind onto research about the architecture of the brain, we organize the text in two sections for each major aspect of the architecture: one for the mind and one for the brain.

### 2.1. Domain-Specific Mental Processes 

Domain-specific systems interface the mind with different aspects of the environment. Psychometric, cognitive, and developmental research agree on five domains of thought: verbal, quantitative, spatial, causal, social, and lingustic. All domains emerge as distinct dimensions in various types of modeling performance on cognitive tasks such as exploratory and confirmatory factor analysis, and graph analysis (see Figure 1). These domains have been described and substantiated elsewhere (Carey 2009; Carroll 1993; Case 1992; Case et al. 2001; Demetriou and Bakracevic 2009; Demetriou et al. 1993, 2002; Demetriou and Kazi 2006; Gelman 2003; Golino and Demetriou 2017). They will only be outlined here for the present purposes.

Each domain involves: (i) perception-based core processes and (ii) representation-based mental operations. Table 1 summarizes the composition of domains across these two types of processes. Core processes are rooted in perception. They frame how meaning is initially made of objects and they appear early in life, if not at birth. Color perception in categorical thought (Eguchi et al. 2017), subitizing in quantitative thought (i.e., automatic recognition of the numerosity of small sets) (Dehaene 2011), depth perception in spatial thought (Hermer and Spelke 1996), perception of causality in causal thought (Saxe and Carey 2006), and face perception in social thought (Simion et al. 2001) are present around birth to ignite the construction of mental operations and concepts. With development, core processes develop into domain-specific operations, such as sorting in categorical reasoning, mental rotation in spatial reasoning, arithmetic operations in quantitative reasoning, isolation of variables in causal reasoning, and moral reasoning in social thought (Demetriou and Spanoudis 2018). Basically, domain-specific operations are generalizations of core processes. They include systems of actions and representations integrating variations in the functioning of core processes over occasions and they may fill in lags of information when core processes do not suffice. The functioning of core processes and operations generates knowledge and beliefs about the domains concerned.

### 2.2. Brain Networks for Domain-Specific Mental Processes

**Figure 2:**
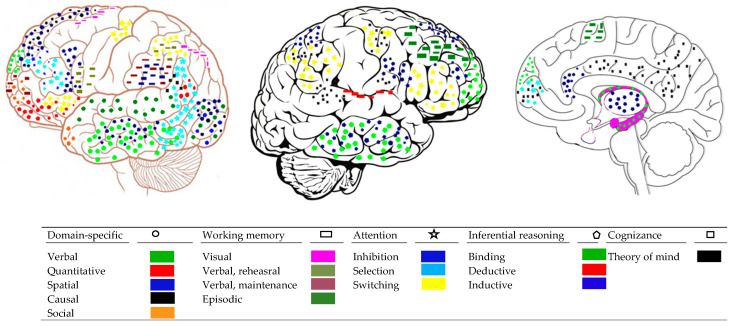
Brain regions associated with different mental processes. Note: Shape indicates systems of processes and color indicates specific processes within a system.

The first question in the introduction asked if the architecture of mind suggested by psychological research is reflected in the organization and functioning of the brain. This seems to be the case when we come to core processes and domain-specific operations. Research suggests that there are entry nodes in the sensory cortices corresponding to each of the core operations above which are highly modular (Yue et al. 2017). Specifically, corticocortical networks display modular organization, alternative processing paths, and highly connected hubs (Zamora-López et al. 2010). Different aspects of visuo-spatial information, such as depth and orientation or gloss, which stand for different core processes in spatial thought, are processed by overlapping regions in the visual cortices (dorsal visual area V3B/KO; kinetic occipital region; (Dövencioğlu et al. 2013; Livingstone and Hubel 1988; Sun et al. 2015)). Discrete quantitative information, such as subitization, and separate numbers are processed by specific brain networks located in the visual and the parietal cortex (Dehaene 2011; Anobile et al. 2016). Perception of causality is served by visual cortices, such as V5 (Rolfs et al. 2013). These cortices, in the right hemisphere, register the actual physical relations between causally interacting objects, abstracting causality from rapid interactions between moving objects (Roser et al. 2005). Finally, in social understanding, the inferior occipital gyrus seems to be the entry node for face recognition (Zhen et al. 2013). Figure 2 illustrates the main regions associated with each domain. 

Information from entry nodes is sent to more general purpose integrative cortices that may be associated with mental operations (Taylor et al. 2015; Yue et al. 2017). Specifically, relational processing underlying mental operations is carried forward, reaching the associative parietal and integrative frontal cortices and temporal cortices, when language is involved. Processing of verbally expressed categorical information is associated with the entire superior temporal gyrus, which is related to language understanding and it thus analyzes the “object” properties of auditory signals (Galaburda 2002). Implementing already acquired expertise in object categorization, regardless of their identity, involves the fusiform gyrus in the inferior extrastriate visual cortex (Gauthier et al. 2000). Mental rotation, an integrative operation of spatial thought, activates the right posterior parietal lobe, centered on the intraparietal sulcus (B7; it is noted that there are inconsistencies in the literature in naming brain areas; some researchers use the Brodmann system for naming areas in the cortex and others use anatomical terms specifying brain areas in 3-dimensional space; in this paper, we kept the original naming system used by each article to avoid misspecification of areas; however, when the Brodmann system is used we also use the anatomical terms in parenthesis to minimize confusion); three-dimensional rotation also activates the right dorsal premotor cortex (Harris et al. 2000; Kawamichi et al. 2007). Categorical encoding of spatial positions activates more the parietal cortex within the left hemisphere than within the right. However, encoding of the same relations in spatial coordinate terms caused more activity in medial temporal cortex and dorsal striatum (Baumann et al. 2012). Number relations, such as increase along the number line, are processed in the inferior parietal cortex, the angular gyrus (BA 39) in particular, and the intraparietal sulcus. Notably, the horizontal segment of the intraparietal sulcus (hIPS) seems to serve the symbolic representation of number by interlinking neural populations directly indicating number, such as dots, with neural populations coding for number symbols, such as digits and number words (Piazza et al. 2007). The frontal cortex is also involved (Dehaene 2011; Nieder and Dehaene 2009). Inference about causal relations is associated with activity in the left hemisphere, where causality from contingencies between events is inferred, such as first pressing a switch and then observing its effect, i.e., the onset of a light (Fonlupt 2003; Roser et al. 2005). Finally, face encoding involves, in addition to occipital regions, right hippocampus and left and right prefrontal cortex, showing similarities with expert categorization (Haxby et al. 1996; Kanwisher et al. 1997; Zhen et al. 2013).

#### 2.2.1. Mental Systems for Attention and Working Memory

There is general agreement that a complex attention system enables the organism to focus on stimuli of importance according to a goal and control interference from surrounding irrelevant but prominent stimuli. The attention system involves three fundamental components: an alerting system controlling arousal and vigilance; an orienting system related to the prioritization of input through selection of modality or location; and a top-down system that handles conflicts and is related to executive control. The current discussion about mastering executive control in development basically refers to the acquisition of self-directed actions that may enable the individual to place the three attention components under intentional control (Zelazo 2015).

Attention systems deliver their content to retention processes operating in the sake of integration of current information with past experience, current action, and planning. There are three types of retention processes: (i) modality-specific short-term storage processes (STS) related to the perceptual origin of information, such as acoustic and visual storage; (ii) executive processes, representing the currently active mental goal controlling information search and integration; together, these two types of processes are known as working memory; (iii) an episodic integrator capable of integrating information across modes into unitary episodic representations preserving the time and subjectivity of experiences (Conway 2005). Strictly speaking, out of these processes, only STS directly serves retention. Executive and episodic integration processes may actually belong to the other types of systems, such as integrative and awareness systems, which are discussed below. Brain research may resolve this confounding problem (Baddeley 2012; Cowan 2010; Pennartz 2015).

#### 2.2.2. Brain Systems for Attention and Working Memory 

##### Attention

Each of the mental attention systems above is served by a different network in the brain (Posner and Rothbart 2007; Petersen and Posner 2012). The alerting system is based in the brain stem system and the thalamus. This is a bottom-up network that projects to the frontal and the parietal cortices. The activation of this network renders one ready for awareness but it does not by itself generate awareness (Dehaene et al. 2017). The orienting system is served by the frontal eye fields (FEF) and parietal areas and frontal areas. Moreover, this system seems to involve two discrete functions: one serving top-down visuospatial selection [intra-parietal sulcus (IPS)/superior parietal lobule (SPL) and FEF] and one serving bottom-up reorienting [temporoparietal junction (TPJ), i.e., inferior parietal lobule (IPL)/superior temporal gyrus (STG) and ventral frontal cortex (VFC), i.e., inferior frontal gyrus (IFg)/middle frontal gyrus (MFg)]. This system may be the gate to conscious awareness discussed below. Finally, the inhibition and executive system involves two networks, a frontoparietal network (precuneus, middle cingulate cortex (mCC), dorsolateral prefrontal cortices (dlPFC), dorsal frontal cortices (dFC), IPS, and IPL) serving moment-to-moment task monitoring; the second is a cingulo-opercular network: dorsal anterior cingulate cortex (dACC)/medial superior frontal cortex (msFC), anterior prefrontal cortex (aPFC), anterior insula/frotal operculum (al/fO)] serving task maintenance. This system may yield awareness of the objects of current thought (Dehaene et al. 2017) (see Figure 2).

##### Working Memory Processes

Several brain structures and networks serve working memory. Jonides et al. (2005) suggested that STS is mediated by the same structures that process perceptual information. That is, when perceptually activated, information-specific brain modules retain information and relay it for further processing to a higher-level system that integrates over event sequences within the same module or across modules, such as integration of visual and acoustic information: phonological storage is associated with a bilateral anterior-prefrontal/inferior parietal network; visuo-spatial storage is associated with posterior parts of the superior frontal sulcus and the entire intraparietal sulcus. Some brain regions, such as the frontoparietal systems associated with attention are activated during processing of both, verbal rehearsal and the visuo-spatial transformations, such as spatial sequencing and rotation (Baddeley 2012; Gruber and von Cramon 2003; Repovš and Baddeley 2006). 

Hippocampus has a special role in episodic integration, specializing in the explicit representation of novel information and temporal and spatial order. It temporarily binds together several distributed areas in the cortex, representing various kinds of information (Conway 2005; Meeter et al. 2005; Sun et al. 2020). In fact, it is itself differentiated so that different cell assemblies in it serve different aspects of working memory, such as (i) retaining information received from visual or phonological cortices; (ii) specifying arrangement information, such as object location and sequential order (Lisman 2005); and (iii) delivering information to the frontal cortex for relational integration (Friedman and Goldman-Rakic 1988; Rubin et al. 2017). 

These retention networks interact with attention networks that may ensure attention control in its various manifestations: they focus on and maintain the currently relevant items (e.g., ventral and/or posterior prefrontal cortex (PFC) maintains the currently relevant items (Ranganath and D’Esposito 2005)); construct multimodal object representations (e.g., medial temporal and inferior temporal regions); and shift between objects, if needed i.e., the inferior frontal junction (IFJ). This later function causes the attentional bottleneck because it can retain only one rule at a time (Vergauwe et al. 2015). Finally, response selection correlates with activation in medial and ventrolateral pre-frontal regions (Cowey 1996; Gruber and Goschke 2004; Rubin et al. 2017; Yoon et al. 2008). When selection is affected, processing is delivered to the integrative and awareness processes discussed below. These findings indicate that executive processes belong to integrative or awareness systems rather than to retention as such.

### 2.3. Integrative Mental Processes

In psychometric theory, general intelligence, g, stands for these processes which are thought to significantly account for individual differences on any cognitive task. It is beyond the scope of this article to embark on the long-standing debate about the nature of g. Interpretations vary enormously. Inductive and analogical reasoning (Spearman 1904; Gustafsson 1984), processing speed (Jensen 1998), working memory (Cowan 2010; Kyllonen and Christal 1990), and executive control (Blair 2006) were invoked as the core of g. However, none of these processes correlates with g higher than 0.5, indicating that g cannot be reduced to any one of them. In response to this state of affairs, several scholars stripped g of any distinct psychological process, claiming that g is an algebraic consequence of the interaction between specific processes (van der Maas et al. 2017), perhaps with some of them being more powerful than others in driving the interaction, such as executive attention that is shared by most cognitive tasks (Kovacs and Conway 2016). Our research showed that all of these processes, together with awareness, are additive components of g, capturing 98% of variance in any domain (Demetriou et al. 2017, 2018; Makris et al. 2017) (see Figure 1).

The common core of the mind must be general enough to cut across all four types of systems (domain-specific, attention and retention, inferential, and awareness). This core must have indentifiable properties at both the psychological level and the neuronal level going beyond the specifics in each of the four types of processes. To achieve this aim, we suggest a mechanism that may look for and extract patterns from information or representations, inter-relate them and generate new patterns or representations that may be more inclusive and accurate in representing the situations of interest. This mechanism involves three inter-dependent processes: (i) representational Alignment; (ii) Abstraction; and (iii) Cognizance. This is the AACog mechanism. Alignment is a “search, vary, and compare” relational mechanism interlinking stimuli and/or representations together according to current goals; thus, alignment is an executive mechanism of representational integration, involving shifting between representations or between representations and responses (Miyake and Friedman 2012). Abstraction spots or induces similarities between patterns of information based on perceptual or semantic properties which feed further alignment processes. Cognizance is part of reflexive consciousness (Dehaene et al. 2017), which includes awareness of one’s own mental processes (“I know that I see by my eyes”), knowledge (“I know I saw apples before”), and abilities (“I can remember what I saw before”). It draws on self-monitoring, reflection, and metarepresentation, allowing explicit encoding of abstractions and reflections on them into new representations (see Figure 1). 

To function, AACog, requires attention and working memory processes but it goes beyond them in that it processes the contents of attention and memory. Cognizance is required because it extends experience beyond the here and now. It may arise any time automatic pattern abstraction is not possible or alignment goes wrong. Choosing between stimuli or actions turns the “mind’s eye” towards both, thereby bringing them into the focus of awareness. Thus, cognizance brings a particular mental content in focus and shifting between mental contents. So defined cognizance allows feedback loops where cycles of alignment of representations, abstraction of relations, and inference may become the object of further alignment and abstraction until a final conclusion is reached, often projected into a new concept. From an adaptational point of view, cognizance reflects the mind’s need to re-work experiences in order to select among them, stabilize whatever appropriate, and integrate with past experience. Thus, it is the basis of mental time-line where past, present, and future are aligned. 

Cognizance processs may bring specific mental processes themselves in focus. For instance, trying to align stimuli may bring memory into focus (because some information is lost in the process); abstracting relations may bring inference in focus. Metarepresentation is a fundamental component of cognizance in that it encapsulates the results of abstraction and alignment into new mental units and encodes them into new representations that may be retrieved as such (e.g., a new word, a new personal mental image, or a new arbitrary symbol). A series of studies suggested that cognizance is an integral component of general intelligence (Demetriou and Kazi 2006), it recycles in development with the cognitive processes dominating in each cycle (Demetriou et al. 2017), and it drives transitions across developmental cycles (Kazi et al. 2019).

AACog integrates (Spearman 1904) eduction of relations (abstraction) and correlates (alignment) underlying g, Piaget (2014) reflective abstraction and equilibration, and reasoning and consciousness that dominated in post-Piagetian developmental research (Scott et al. 2014). Inference is a fundamental tool in the service of the AACog mechanism. Early in development, Bayesian inference induces (first-order) relations based on statistical regularities (Tenenbaum et al. 2011). Later, inductive, analogical, and deductive reasoning construct relations at various levels of abstraction based on extrapolation of rules (induction) or deduction from rules about truth and consistency already available (Demetriou et al. 2017). 

#### 2.3.1. Brain Processes Serving Intergative Mental Processes

##### AACog

The neural analogue of the AACog mechanism would include a sequence of neural networks that would map onto the Alignment, Abstraction, and Cognizance processes of this mechanism. Specifically, there should be (i) networks which would first register information or information patterns (alignment); (ii) then there should be networks where these patterns are projected in order to be associated with relevant information (abstraction); (iii) finally, these relations would be made available to another network which makes final choices (an answer, a solution, or a conclusion) which may be a combination, reduction, or variation of representations considered; (iv) a parallel network here may spot application problems for the networks above and provide evaluative feedback that allows optimization of choices (cognizance). In the process, any of these networks may call upon networks for support, such as retention or inferential networks networks (e.g., inductive or deductive reasoning). Notably, recent research suggests that focusing on semantic abstract information, such as the notion of the apple in Judeo-Christian tradition, recruits a series of networks related to the physical haracteristics of apples (their color, shape, etc.); this interaction between networks indicates a brain search and alignment processes that carries information at a higher level where it is integrated as an abstraction serving a general semantic category (Fairhall 2020).

This cascade of networks has been recognized several times. Jung and Haier (2007) proposed the parieto-frontal integration theory (P-FIT) as the brain analogue of g. According to P-FIT, information is first registered and processed in regions of the cortex activated by different types of sensory information, such as visual (BA 18, 19) or auditory cortex (BA 22). Therefore, the sensory areas involved in the P-FIT model may be more related to the domain-specific processes represented by the present model, such as categorical, spatial, or quantitative core operations. From there, information is fed forward to several regions in the parietal cortex (BA 7, 39, 40), which primarily carry out elaboration, association with past knowledge or action, and abstraction. Therefore, the parietal areas of the P-FIT model seem related to the abstraction and alignment processes in AACog. Then, these regions interact with frontal regions (BA 6, 9, 10, 45–47) in search of alternative solutions to the problem at hand. These appear related to cognizance in the AACog model. Based on recent evidence, Haier (2016) expanded P-FIT to include further regions in the medial frontal cortices, such as the anterior cingulate (BA 32) (engaged in response selection by inhibiting alternative responses) (Gruber and von Cramon 2003; Klingberg 1998); the posterior cingulate and subcortical structures, such as the caudate nucleus are also related (Basten et al. 2015). 

##### Reasoning

Interestingly, networks associated with inductive and deductive reasoning overlap but are not identical with the networks above. Specifically, simple inference based on Bayesian probabilistic covariation relates to activation in the left cerebellum and adjacent visual cortex; this network seems to integrate relations between movement and visual information (Ide et al. 2013; Zheng and Rajapakse 2006). Bayesian abstraction of functional covariation between events involves, additionally, anterior cingulate and prefrontal cortex, which is always present in relational thought (Ide et al. 2013; Wendelken et al. 2015). Inductive reasoning activates the frontal gyrus (serving integration) and the right insular cortex, serving salience detection and switching between large scale networks. This system activates attention and working memory networks to ensure focusing on and representing the salient representation selected (Menon and Uddin 2010) (see Figure 2). 

Deductive reasoning activates several networks serving different tasks at different stages of the inferential process. Specifically, content based propositions activate temporal (BA 21, 22, serving language processing) and frontal regions (BA 44, 8, 9, serving integration). Formal propositions activate occipital regions (BA 18, 19), suggesting construction of visual mental models of the relations implied by the formal propositions, left parietal (BA 40, building associations), and bilateral dorsal frontal (BA 6), left frontal (BA 44, 8, 10), and right frontal (BA 46) regions, serving integration, evaluation, and selection (Goel et al. 2000; Goel and Dolan 2000, 2001). Other studies suggested activation of the right superior parietal lobule (serving associations between concepts), the thalamus (relaying information between systems), and the right anterior cingulate (serving selection of competing responses) (Osherson et al. 1998). 

Vendetti and Bunge (2014) proposed that three regions are central to analogical or deductive reasoning. The first, located in the IPL, represents specific rather than general relations and it scales with the number of relations to be considered. This seems related to abstraction as it makes first-order relations available to the second region, rostrolateral prefrontal cortex (rlPFC). This region aligns first-order relations and abstracts second-order relations, comparing and integrating mental representations over common relations running through them. The third region, dlPFC, provides a supporting role, enabling performance monitoring, interference suppression, response selection, and manipulation of items in working memory. The second region does not but the third does scale with difficulty. Thus, this relates to cognizance more than with the other AACog processes. Age-related increases in myelination of the left rlPFC-IPL tract, but not the corresponding right tract, predict reasoning development (Wendelken et al. 2015).

##### Cognizance

There is no consensus about the brain bases of consciousness in general and cognizance in particular. Some scholars maintain that consciousness is associated with specifically dedicated brain structures (Crick and Koch 2005); others claim that it is an emergent dynamic condition reflecting the co-activation of several networks (Baars 1993). It is beyond the concerns of this paper to embark on this discussion. From the point of view of the present concerns, there is strong evidence that some regions and networks are always involved in cognitive awareness. Some aspects of attention, such as selective attention, or focusing on mental representations, as in mental rehearsal, are linked to cognitive awareness because they operate as the gate between unconscious and conscious mental functioning. 

However, explicit awareness of mental states always involves the prefrontal cortex. fMRI studies suggest that introspection discriminating correct from incorrect performance involves the prefrontal cortex. Fleming et al. (2010) showed that introspective ability was correlated with gray matter volume in the anterior prefrontal cortex. Moreover, interindividual variation in introspective ability was correlated with white-matter microstructure connected with this area of the prefrontal cortex. Experimental inactivation of the prefrontal cortex handicaps metacognitive judgements about performance on a task but spares processing for the task. Rounis et al. (2010) used transcranial magnetic stimulation to depress activity in the dorsolateral prefrontal cortex as participants performed a visual discrimination task. They found that participants were able to perform the discrimination task but they were not able to discriminate between correct and incorrect stimulus judgements. In fact, some theorize that metacognitive monitoring is one of the major evolved competences of the prefrontal cortex (Carlén 2017; Dehaene et al. 2017) (see Figure 2).

How does this mechanism operate? According to the global workspace model (Baars 1993), a particular object of thought would reach consciousness and become the object of explicit awareness when the corresponding neural population is mobilized by top-down attentional amplification into a self-sustained brain-scale state of coherent activity that involves many neurons distributed throughout the brain. In its modern version, “local specialized cortical processors are linked, at a central level, by a core set of highly interconnected areas containing a high density of large pyramidal neurons with long-distance axons (in parietal and prefrontal areas). At any given moment, this architecture can select a piece of information within one or several processors, amplify it, and broadcast it to all other processors, thus rendering it consciously accessible and available for verbal report”. (Mashour et al. 2020). It is as though this particular content seizes the whole brain for some time and is made available to various processes, such as categorization, quantitative estimation, long-term storage, through the long-distance connectivity of these top-down amplified ‘workspace neurons’ (Sergent and Naccache 2012, p. 102). Evidence suggests that two regions are causally crucial in this process: mPFC and midline parietal cortex (mPC). Inactivating them by anesthesia annihilates awareness (Boly et al. 2013). Below, we examine how networks communicate.

Overall, relating, abstracting, and inferring is a stepwise process activating overalapping cortices. This processes evolves over a sequence of steps where information is registered and represented by particular networks, often domain-specific (these may be in visual, acoustic, or cerebellar networks). The abstraction of common patterns is sent to other networks which are sensitive to the patterns (IPL). Then inferential integration of the patterns in terms of inductions or deductions are delivered to other networks (in the PFC) which “impose” connections, such as extrapolations of relations induction, or conclusion selection in deduction. These later processes involve awareness and evaluation, where content-laden networks become concurrently available to a cognizance template for inspection and evaluation based on criteria of logical validity or truth. 

#### 2.3.2. Communication between Brain Networks

So far, we talked about interactions between brain networks related to cognitive processes, but we did not specify the neuronal processes underlying these interactions. The second question stated in the introduction was concerned with these neuronal processes. It seems that these processes are expressed in electrodynamic activity taking place at various frequencies. Brain rhythms are periodic oscillations in excitability of groups of neurons as reflected in EEG activity. Rhythms vary from very low (i.e., 0.05 Hz frequency) to very high (200–600 Hz). Perceptual and cognitive activity is mainly expressed into delta (1.5–4 Hz), theta (4–10 Hz), alpha (7–13 Hz), beta (10–30 Hz), and gamma rhythms (30–80 Hz) (Buzsáki et al. 2013). 

It seems that different rhythms are associated with different brain regions, serve different functions, and they are hierarchically organized during cognitive processing. Specifically, alpha and beta oscillations arise in the visual cortex and the thalamocortical system and serve visual perception and related sensory-sensory and sensory-motor coordination (Thut and Miniussi 2009). Beta oscillations also arise in the motor cortex and transfer messages to the motor systems. Gamma oscillations serve multiple purposes. They are associated with local computation but they are also involved in selective and flexible coupling of neighboring and distant regions, such as the hemispheres, to integrate correlated information. Feature binding, perceptual closure, focus of attention, and maintenance of contents in working memory are closely associated with increased beta- and gamma-band oscillations and enhanced synchronization (Irrmischer et al. 2018). Theta oscillations originate in prefrontal–orbitofrontal regions and propagate caudally; they also arise in the hippocampus. Slower rhythms, such as theta rhythms, can reset and modulate the power of faster rhythms running local computation in different cortical areas, channeling it to current computational needs. These rhythms constitute the basic components and syntactic rules of brain language (Buzsáki and Watson 2012; Buzsáki et al. 2013).

For instance, in a sequence of items presented in working memory tasks, such as successive letters or digits, items are encoded by high frequency rhythms, such as gamma oscillations emerging in the hippocampus. Gamma oscillations may be gating only the most excited items to fire, synchronize spikes, and delimit items by creating pauses between them (Lisman and Jensen 2013). These stand for the neural letters of thought. These letters are combined into “neural words” and neural sentences according to a specific rule, such as their presentation order, which is encoded by lower frequency rhythms, such as theta oscillations, emerging in the prefrontal cortex (Buzsáki and Watson 2012). Perhaps, the sequence of gamma oscillations in the hippocampus stands for STS; the sequence of theta oscillations in the prefrontal cortex stands for the executive processes, elevating STS into working memory (Jensen and Lisman 2005). Lisman (2005) suggested that working memory capacity equals the number of gamma cycles (standing for individual stimuli) that can go within a theta cycle. Thus, integrated gamma/theta cycles stand for a brain code for storing multiple items in working memory (Segneri et al. 2020). 

Others suggested that theta activity is the fundamental integrative mechanism of the brain that coordinates different types of information expressed into other brain rhythms (Sauseng et al. 2010). Maguire et al. (2010) showed that theta power increased over right frontal areas when processing thematic relations between words (e.g., leash-dog); alpha power increased over parietal areas when processing taxonomic relationships (e.g., horse-dog). Along this line (Clarke et al. 2018) showed that frontal theta and posterior alpha/beta oscillations play a key role during associative memory formation. Frontal midline theta oscillations were stronger at the beginning of learning and declined with increasing strengthening of stimuli-response associations. At this early phase, posterior alpha and low-beta oscillations decreased and they increased with strengthening of associations. This pattern suggests that changes in theta oscillations reflect changes in the control needs of the learning task (e.g., better maintenance of cue information, thereby supporting better encoding of the stimulus-response relation); changes in the alpha/beta oscillations reflect changes in the learning attained (i.e., establishment of the stimulus-response relation) and ensuing changes in inhibition needs. In line with these findings, Segneri et al. (2020) recently demonstrated experimentally tthat changes in the amplitutes of theta oscillations force theta-nested gamma oscillations to chane proportionally in the range of 1 to 10 Hz. Noticeably, (Reinhart 2017) demonstrated recently that experimental enhancement by means of transcranial alternating current stimulation of the coordination of theta oscillations in two different regions of the prefrontal cortex (medial frontal cortex and lateral prefrontal cortex) improved executive control. Experimental disruption of this coordination weakened executive control. These findings indicate that theta oscillations are brain carriers of executive control. Along the same lines, Jaušovec and Jaušovec (2014), showed that transcranial alternating current stimulation over the left parietal region but not the left frontal region resulted in enhanced working memory performance. These findings indicate that different brain regions use the theta band to exert control on other regions. 

In conclusion, the research reviewed above suggests that there are several (overlapping) networks in the brain sub-serving each one of the various systems of the mind; in fact, various brain rhythms are used in the service of the interactions between these networks. Below, we will discuss the development of these networks. 

## 3. Development 

### 3.1. Generally Accepted Assumptions about Cognitive Development

Ever since Baldwin (1906) and Piaget (1970), students of intellectual development described a sequence of four stages in cognitive development spanning from birth to adolescence. This classic stage sequence has been extensively investigated and redefined by several scholars, each focusing on a different aspect of intellectual development, such as logical reasoning (Piaget 1970), working memory (Pascual-Leone 1970), relational complexity (Halford et al. 1998), and executive control (Case 1992; Zelazo 2015). Despite differences in emphasis or language, this scholarship converged on several recurrent findings:

Major transitions in the nature of representation or concepts occur around the same age: at 2, when there is a representational spurt culminating in language attainment; at 7, when there is a spurt in mental cohesion and flexibility, culminating in learning to integrate over representational systems to build new complex skills, such as reading and school arithmetic; and 11 years, when there is a spurt in dealing with abstract ideas and using related arbitrary symbol systems, such as algebra (Demetriou et al. 2020). There may be variations across domains, but these changes are fairly independent of tasks or methods of analysis. To integrate over theories and empirical findings, we suggested a four-cycle developmental sequence, with two phases in each. New representations emerge early in each cycle and their integration dominates later. The four cycles differ in their dominant unit of representation. They are as follows: (1) interaction episodes and modality-specific memories; (2) language (words) and mental images; (3) mental blueprints and action scripts defined by rules, sometimes symbolized by artificial symbol systems such as numerals; (4) canonical principles prescribing acceptable possibilities, sometimes symbolized by highly idiosyncratic symbol systems, such as notation in different sciences. 

The emergence and consolidation of a new unit of representation in each cycle sparks the emergence of new representational systems which mark transition across cycles: (i) speaking at 2; (ii) reading, writing, and arithmetic at 6; (iii) abstract idiosyncratic symbolic systems, such as advanced notation in mathematics, science, or arts, at 11 years. New symbol systems open new possibilities for the representation of increasingly complex states of the world. Thus, representations in successive cycles differ in resolution, predication, symbolizability, connectivity, and cognizability. This is basically the reason why cognitive constructs become increasingly abstract, complex, and logical with development. The term “cycle” over “stage” or “level” emphasizes that development is a continuous process systematically transforming representations and mental processes rather than a succession of steady states. The ages above are modal ages capturing the development of the majority of individuals. There may be both intra- and inter-individual differences in the rate of development across domains or processes, indicating continuity rather than discontinuity in development (Siegler 2016).

These cycles are outlined below with an emphasis on executive control, cognizance, and reasoning. It is emphasizied that developmental priorities change across cycles. Specifically, different mental processes dominate in successive developmental cycles depending upon the functional and adaptive needs of each phase. Overall, g gradually shifts from executive to inferential and self-awareness processes. The cognitive profile of different age phases constrains what can be learned and what problems can be solved (Demetriou and Spanoudis 2018; Demetriou et al. 2017, 2018). The reader may consult other sources for a discussion of the relations between this sequence and other theories (Demetriou and Spanoudis 2018; Demetriou et al. 2017, 2018). 

### 3.2. Developmental Cycles in Cognitive Development 

Episodic representations are blueprints of actions and experiences preserving their spatial and time properties. Interaction control is the major developmental priority of this cycle: infants must learn to interact with objects and persons through different modalities, capitalizing on the affordances of the environment and their own bodily possibilities and skills (Thelen and Spencer 1998). Interaction with the environment generates episodic representations. There is no executive control until 4–5 months of age; directed attention and inhibition appear systematically in the 6–9 months window (Holmboe et al. 2017). By this age, attention-based actions may be initiated by interesting stimuli or action sequences may be restoted. Along with the second year of life, recurring event sequences are aligned into episodic representations yielding mental blueprints of action (e.g., follow, manipulate, recover, and coordinate objects and actions) and inference (Carey 2009). Awareness is still minute and transient. However, by 15–18 months, infants show awareness of actions performed; they seem to demonstrate an executive sequence where past actions are intertwined with perceptions and current actions, indicating awareness of the contents of activity (Dehaene et al. 2017). Reasoning as such does not exist in this cycle (Xu 2019). Episodic reasoning involves reciting episodic representations or actions in sequences predating reasoning schemes such as conjunction (e.g., “I put this, and this, and this, all of them”), disjunction (e.g., this one, not this one), or implication (“first this then this) (Demetriou and Spanoudis 2018).

Realistic representations: At the end of the second year, episodic representations are projected into realistic mental representations. These are blueprints of episodic representations distilled from initial spatial and time properties, associated with symbols, primarily mental images and, increasingly, with words. Control of attention is the major developmental priority of this cycle. Holding representations active and in focus so that information in the senses is encoded, processed, chosen or ignored, according to its relevance to the currently focused goal so that an action sequence may implement a mental plan. By the age of 3, children can subject their behavior to the control of a represented goal and implement the actions required, as in go/no go tasks (Diamond 2013; Zelazo 2015). In the second phase of this cycle children demonstrate representatonal awareness in several domains. Thus, attention control and representational awareness are major markers of g in the 4–7 years period (Demetriou et al. 2017). Children now understand that perception is a source of knowledge (Spanoudis et al. 2015). In ToM tasks children understand that different individuals may have different representations, depending on what they perceive (Wellman 2014). By the end of the cycle, representational awareness is a crucial factor for learning to read and understand arithmetic (Peters and De Smedt 2018). Reasoning emerges in this cycle but is secondary to attention control and representational awareness. Representations may be aligned into sequences yielding conceptual inductions (e.g., he barks, he is a dog) and “plausible deductions (e.g., “it’s cloudy”; “it will rain”). However, there is no explicit awareness of the logical links between representations. 

Rule-based thought; At 6–8 years, with attentional control established, links between representations begin to emerge; language predication contributes to establishing these links. This complexity presents a new developmental challenge: identify relations between representations and organize them so that they can be called upon efficiently in sake of understanding and interaction. The major task of this cycle is to master the process dealing with relations between representations. Thus, inferential control is the major developmental priority in this cycle. In this cycle, children begin to use rules in solving inductive and deductive reasoning. In inductive reasoning, which is a major marker of g in this age period (Demetriou et al. 2017), children make the relational shift, looking for relations between relations (Gentner 2005). In deductive reasoning, they reach bi-conditional reasoning where modus ponens (p and q; p; q) and modus tollens (p and q; not q; not p) are integrated into a common scheme (Barrouillet and Lecas 1999). However, they still fail logical fallacies. These changes come with cognitive awareness in several domains and flexibility in shifting between them. Children become explicitly aware of inference as a tool for creating knowledge and linking representations. (Kazi et al. 2019). They differentiate between easy and difficult memorization tasks, suggesting awareness of the relation between complexity of representations and learning (Chevalier and Blaye 2016). 

The principle-based thought: Truth control is the developmental priority of principle-based thought. Thus, adolescents begin to command conditional reasoning, resisting logical fallacies, which is a major marker of g in this cycle (e.g., p and q; not p; unknown if q) (Christoforides et al. 2016; Demetriou et al. 2017). The principle-based mind adopts a suppositional stance, viewing realities from multiple perspectives. They also gradually grasp the principles connecting inferential rules (Makris et al. 2017). Early in adolescence, at 11–13 years, adolescents begin to form accurate maps of mental functions and their own strengths and weaknesses. As a result, they evaluate their own performance on cognitive tasks with increasing accuracy. Control in this cycle is based on a cohesion and validation program allowing to co-activate conceptual spaces, evaluate them against each other, and form long-term life plans (Moshman 2004). 

### 3.3. Changes in the Integrative Processes of the Mind

Integrative processes emerge with development. In (Fodor 1975) terms, a Language of Thought (LOT) is not innate; it is gradual output of the reorganization of mental processes in intellectual development. Its basic proto-representational units are generated by the core operators within each of the domain-specific systems and they then evolve into the basic representational units of each cycle (episodic, realistic mental, rule-based, and principle-based representations). The control priority of each cycle defines the syntactic rules of LoT integrating representations in each cycle. In infancy, episodic representations are integrated in the flow of episodic events. Thus, the attention-based transient and fragmented awareness of action episodes generates proto-reasoning experiences, such as recursions (e.g., “and … and … and …”) or choices between events or objects (or … or). However, infants cannot be credited with reasoning because these associations are not represented as such (Xu 2019). The attention control program of the representational cycle adds a representational dimension to the interaction control program of the episodic infant, enabling preschoolers to develop parallel mentally based action plans in addition to stimulus initiated action plans. Explicit awareness of representations and their streaming according to a goal in realistic representational thought yields explicit inductions and pragmatic deals. However, inference is still embedded in realistic sequences rather than deduced. The inferential control program in rule-based representations adds an inferential dimension to executive control allowing to coordinate mental spaces in addition to representational-action inhibition processes. Inferential control in rule-based thought generates awareness of rules, allowing systematic premeditated reasoning which culminates in the deductive reasoning of the adolescent (Barrouillet and Lecas 1999; Reverberi et al. 2012). The truth control program in adolescence adds an epistemological dimension to executive control, allowing control based on a long-term time-scale integrating the past, the present, and the future into a cohesive plan. 

### 3.4. The Role of Cognizance in the Development of Integrative Processes

Cognizance mediates between executive control and reasoning from infancy to adulthood. This mediation is cycle-specific, exerted through the processes underlying the management of representation in each cycle: perception-based aspects of representation in the representational cycle, rule-based inferential processes in the rule-based cycle, and abstract semantic processes in the principle-based cycle. This implies that cognizance is a regulatory process registering representations and participating in their metarepresentation into new more abstract and thus flexible forms. In terms of the AACog mechanism, alignmenents and abstractions come increasingly under the influence of cognizance so that they shift from the local environment of core processes to a kind of mental universalism where rules and principles abstracted in the past may be systematically invoked to evaluate or fill in lags of current representational ensembles. 

In line with this assumption, recent longitudinal evidence showed that cognizance drives developmental momentum of executive and reasoning processes from 4 to 11 years (Kazi et al. 2019). Bottom-up mediation is stronger early in development; in the period from about 4 to 7 years, executive processes, attention control, and working memory in particular, generate awareness of mental processes that is used in managing reasoning. Top-down mediation appears from late childhood onwards; at 8–10 years, awareness emerging from reasoning as such is transferred top-down to executive control, enhancing its scope and flexibility (Demetriou et al. 2018; Spanoudis et al. 2015). 

### 3.5. Developmental Changes in the Brain

The third question asked how brain changes relate with cognitive developmental changes. It is well established that brain undergoes both structural and functional changes in development, in several dimensions, including its sheer volume, condition of the neurons, inter-linking within and between regions, and biochemical functioning, including neurotramitters and electrical activity. These changes reflect a dynamic interplay of simultaneously occurring progressive events, such as increases in neuronal matter and synapses, and regressive events, such as cell death and synaptic pruning (Millán et al. 2018). A crucial prediction of the model of the mental architecture and development above is that these changes in the brain must be connected to both, the various systems specified by the architecture and the developmental sequences specified by the developmental dimension of the model. 

#### 3.5.1. Changes in Brain Structures and Networks

The sequence of cognitive changes above suggest that domain-specific processes mature first; attention control processes start to mature in the second year of life but they culminate as attention control and representational awareness processes from 3 to about 6 years; inductive reasoning processes take off in middle childhood at about 7–8 years and they culminate as deductive logical reasoning in adolescence at 13–15 years. We therefore, expect a pattern of changes in the brain networks associated with each of these processes at the corresponding age windows. That is, one would expect that sensory-motor cortices underlying domain-specific core processes would mature first; then these would interlink with parietal associative cortices and attention control and mentalizing networks; then both sensory and parietal cortices would interlink with frontal cortices underlying integrative inductive and analogical reasoning networks; finally, specific cortices related to self-awareness and self-evaluation may emerge. Below we will first review research related to developmental changes in brain structures and networks. We will then focus on changes in brain functioning.

Total brain size increases drastically from birth to middle childhood, reaching about 90% of adult size by the age of 6. Synaptic density (i.e., the number of synapses/mm^3^) changes exponentially up to the first year: it is about 0.5 × 108 at the fifth gestational month and it increases to 2.5 × 108 at birth to 5.8 × 108 at the end of the first year; it then slows down to 3.5 × 108 at 10 and 3 × 108 at 70 years). In line with our predictions, synaptogenesis and synaptic pruning underlying brain networking differs across brain regions. Overall, slow wave activity, an index of increases in cortical plasticity, starts early in posterior areas and spreads rostrally to the frontal areas through the first two decades of life (Kurth et al. 2010). The various maturation patterns are consistent with the representational and procedural profile of successive cognitive developmental cycles. Specifically, spontaneous patterned brain activity starts before birth. This activity plays an important role in the formation of the brain networks needed for the activation of perception at birth (Sporns 2016; Sporns et al. 2004). Moreno-Juan et al. (2017) showed that prenatal thalamic spontaneous calcium waves are key regulators of sensory cortical area size and that prenatal alteration in inter-thalamic sensory nuclei communication may trigger size adaptation in cortical fields. Hence, acoustic, visual, and motor networks develop fast in infancy, enabling the construction of episodic mental units (Franson et al. 2011).

It is notable that these changes occur in a context where the scaffold of developing brain networking, as indicated by resting state networks, is in place before birth, with rapid neural growth in the last three gestational months (Hoff et al. 2013). This scaffold includes bilateral primary motor, primary visual and extra-striate visual, parietal-frontal and frontal (executive control networks) insular-temporal/anterior cingulate cortices (ACC; salience and shifting networks). Also, the basics of the default-mode network (DMN), including the posterior cingulate cortex (PCC), the medial prefrontal cortex (mPFC), the medial temporal lobes (MTL), and the angular gyrus (AG), is in place since very early but develops throughout childhood. Supekar et al. (2010) found that the PCC component is present from the second week of life. By the age of 7–9 years, functional connections between PCC, mPFC, bilateral MTL, and bilateral AG attain adult levels. Up to this age, there is an excess of gray matter in children which is pruned in favor of white matter in adulthood, indicating stronger structural connections. 

Attentional, associative, executive, and reasoning networks follow a long developmental process extending to early adulthood. Initially, the attentional orienting network is well active by the age of 6–7 months, allowing attention shifting and focusing as a result of changing stimulation in the environment. This network develops throughout the first and the second years of life, allowing episodic executive control that is needed to look for and recover hidden objects at the age of 18–20 months (Thompson et al. 2000). These connections are strengthened throughout childhood. Also, in the period between 3 and 6 years, there is a drastic change (60%–80% percent increase in connecting fibers) in the frontal circuits of the corpus callosum, which sustain vigilance and regulate action planning. This goes well with the finding that in this phase, at 3–4 years, the inhibition network is effectively functioning, rendering the attention control program of the representational cycle possible. Probably these changes are also related to a spurt in visual working memory and overall representational activity, including imitation and attraction to imaginary stories that preschoolers demonstrate. Later, at 6–7 years, there is dramatic change in the callosal isthmus, which supports intraparietal associative and language functions. Also, in the period from 7–11 years, the anterior cingulate gets differentiated from the orienting network and connects to the executive and the associative IP networks (Rothbart and Posner 2015), perhaps related to the conceptual fluency program attained in this period.

Attentional networks continue to develop throughout childhood. According to de Bie et al. (2012), a cingulo-opercular attentional network appeared fragmented into the two components even at age 8 years: the first component starts from the inferior frontal gyrus (IFG) and extends into the middle frontal gyrus bilaterally; the other comprises the cingulate gyrus extending into the medial superior frontal gyrus, bilateral insula/frontal operculum of the IFG, and bilaterally in the superior frontal gyrus, precuneus, and thalamus and cerebellum. These networks integrate into a single network in adults, which also involves the PCC/precuneus in the ventral frontoparietal network. This network relates with goal directed behavior, salience processing, and cognitive flexibility and it may relate to improvements in executive control attained in adolescence. Similar trends were observed in the development of working memory. Scherf et al. (2006) showed that 10–13-year children only minimally relied on core working memory regions when performing a working memory task; instead, they relied primarily on ventromedial regions (caudate nucleus and anterior insula). The working memory network emerged in adolescence but it was diffused (dlPFC, anterior cingulate, posterior parietal, anterior insula), including premotor response preparation and execution circuitry. In adults (>18 years) this network narrowed down into the specialized working memory circuitry which interlinked with performance enhancing regions, including left-lateralized dlPFC, ventrolateral prefrontal cortex, and supramarginal gyrus 

Several studies investigated the development of the mentalizing network (TPJ, superior temporal gyrus (STS)/middle temporal gyrus (MTG), PC and mPFC) associated with ToM and false belief understanding. Specifically, (Wiesmann et al. 2017) explored the white tracts structural connections in this network, comparing 3–4-year old children who succeeded with their agemates who failed on ToM tasks. They found that age related changes in TemporoParietalJunction, MedialTemporalGyrus, PariatalCortex, and MedialPreFrontalCortex and also age-independent streamlines between temporoparietal and inferior frontal regions were related to grasping ToM. Interestingly, controlling for associations of these networks with executive function and language performance showed that some parts of the networks are specific to ToM (the right TPJ, left MTG, right mPFC and right PC) and they are independent of executive function and language. Notably, (Sabbagh et al. 2009), exploring functional rather than structural connections as expressed in the alpha band sequence, they found that basically the same network (dorsal medial prefrontal cortex (dmPFC), right temporoparietal junction (rTPJ), cuneous, inferior temporal lobe) is associated with ToM in 4-year-old children. They theorized that dmPFC performs the computations associated with complex metarepresentational reasoning; the rTPJ performs the highly specific task of reasoning about representational mental states; the cuneus is engaged when reasoning about self-knowledge and it may also provide support via mental imagery when recalling the foregoing events; the development of inferior temporal regions may contribute to the development of the other regions. 

Some parts of this network [lateral prefrontal cortex (lPFC) and medial posterior parietal cortex (mPPC)] are also associated with reasoning as such. Wendelken et al. (2015) found systematic changes in brain networks associated with first- and second-order reasoning. Specifically, in the 7–10 years cycle, the IPL-rlPFC network is activated but it undifferentiably processes both first- and second-order relations. This differentiation occurs in several phases. In the 6–8 years phase, reasoning development goes with weakening of connectivity between the dlPFC and the ventrolateral prefrontal cortex (vlPFC). In line with our findings about the recycling of relations between speed and working memory, on the one hand, and reasoning, on the other hand, in the 6–8 years phase, changes in speed mediated changes in reasoning ability, suggesting that entering the cycle of rule-based reasoning is associated with differentiation between dlPFC and vlPFC, causing faster processing. In the next phase, in 9–11 years phase, the rlPFC dominated and it was coupled with the right rlPFC. Notably, in this age phase, changes in working memory mediated changes in reasoning, suggesting that consolidation of rule based-reasoning is associated with the consolidation of the relevant brain network which is expressed in working memory expansion. In the 11–14 years phase, left and right dlPFC as well as dorsomedial PFC were more strongly engaged in processing second-order rather than first-order relations. That is, in this phase, the second pole takes its primary function in processing second-order relations. In the 15–18 years phase, left rlPFC and bilateral IPL were engaged in processing second-order relations as contrasted to first order relations. It seems that cortical reorganization within the IPL leads to greater efficiency in processing first-order relations, thereby reducing relational processing demands within rlPFC. Notably, Mackey et al. (2013) found that training in reasoning strengthened the connections between the rlPFC and the IPC but also between the IPL and the striatum. One may see that changes in relations between brain networks and reasoning from 7–15 years coincides with the cycles of rule-based and principle-based thought. 

Changes in other networks apparently related to reasoning develop from middle childhood to adolescence. Wendelken et al. (2017) showed longitudinally that increases in structural connectivity between the rlPFC and IPL are fastest in middle childhood, with a maximal rate of increase at age 7 years; these structural changes preceded functional changes between these regions, which started in late childhood and peaked at age 13 years. In fact, earlier increases in structural connectivity predicted later increases in functional connectivity. Also, some of the changes in structural and functional connectivity were related to changes in reasoning: increases in structural frontoparietal connectivity were strongly related with reasoning development in the period from 6 to 12 years. Therefore, improvements in structural connectivity in this network was related to changes in reasoning ability during the age period of rule construction; improvements in functional connectivity concurred in adolescence, when principle-based reasoning develops. However, development goes beyond adolescence. Marek et al. (2015) showed that the networks linking the ventral and medial prefrontal cortex to limbic and temporal regions continue to change into young adulthood. It seems that the top-down control network is fully established only in late adolescence and early adulthood.

Networks are more diffused early in development. Güler and Thomas (2013) used event-related fMRI to compare brain activation at 8–9 and 12–13 years when learning to memorize and recall unrelated pictorial pairs (e.g., hat-giraffe, balloon-window, etc.). There were some clear differences in both encoding and recall. Younger children recruited wider regions in the right dorsolateral prefrontal and right temporal cortex compared to adolescents during successful encoding of the pairs. However, during successful recall, adolescents recruited wider regions in the left ventrolateral prefrontal and left inferior parietal cortex compared to children. These results suggest that the prefrontal cortex is involved during both encoding and recall but the exact activation changes in development. Younger children need more regions to bilaterally sustain encoding. With age, building relations becomes more efficient and this is reflected in activating special dedicated regions; thus, encoding becomes more focalized because it is more efficient. 

#### 3.5.2. Cycles in the Functional Aspects of Brain Development 

The patterns discussed above would imply corresponding patterns in the development of brain rhythms. Specifically, alpha and beta rhythms, emerging from early maturing sensory and motor cortices should mature before rhythms, such as gamma and theta, emerging from cortices maturing later. Gamma rhythms, which are important in the integration of widely distributed brain modules, including thalamocortical and corticothalamic networks involved in control and inhibition, should accelerate in the 2–6 years phase, when representations spurt and become aligned. Finally, delta and theta bands should peak in late childhood and adolescence, when top-down control culminates. The evidence below appears consistent with this expected pattern. 

Notably, brain changes tractable in EEG recordings appear to match the intellectual cycles outlined here. Thatcher (1992, 1994) and Thatcher et al. (2005) found that electroencephalographic coherency, which reflects changes in connections within and between regions, develops in growth spurts that are nearly identical to the time frame of the developmental cycles described above: four cycles extending from early childhood onwards occur at approximately 1.5–5 years, 5–10 years, and 10–14 years of age, with growth spurts occurring at the ages of 6, 10, and 14 years. Hudspeth and Pribram (1992) reported changes in relative power indices across the brain occurring in stages almost perfectly matching those presented by Thatcher and the cycles proposed here (1–6, 6–10.5, 10.5–13, and 13–17 years). Epstein (1986) showed that spurts in total EEG energy in the alpha band (8–13 Hz) coincide with the first phase of the cycles presented here: They occur at the age of 2–4, 6–8, 10–12, and 14–15 years. 

At a finer level, changes in different rhythms seem to align with the predictions above. Theta activity is present from infancy. Bosseler et al. (2013) showed increases in theta rhythms at the age of 12 months as a response to native syllables, which was not observed in 6-month-old infants or adults. This was interpreted to imply that by this age children recognize their native language and they have their attentional network engaged to process a meaningful sound pattern. Overall, however, there seems to be a shift from low frequency power (<6 Hz) to higher frequency power (>6 Hz) from infancy to early childhood. Specifically, it seems that there is a peak of alpha power at 6–10 Hz at 10 months of age capturing central sensory-motor activity. This band reaches a limit at 9 Hz at the age of four years (Marshall et al. 2002). 

Gamma rhythms emerge in early preschool age and peak in middle childhood. Benasich et al. (2008) found that there is a large increase in gamma power from 16 months to 36 months of age. This increase was systematically correlated with various aspects of language learning, such as auditory comprehension and speech production, and cognitive abilities such as attention shifting and inhibition. Moreover, changes in gamma power over this period clearly differentiated between children with a family history of language impairment from children free of such history: gamma power grew stronger with age in this latter group of children. Notably, these researchers showed in a longitudinal follow up of their sample, that gamma power in the 2–3 years period strongly predicted language (syntax comprehension) and cognitive development at 4–5 years of age (Gou et al. 2011).

Changes in low band control rhythms appear to vary with developmental phase, as expected. Specifically, Orekhova et al. (2006), showed changes in theta and mu rhythms from infancy (6–12 months) to preschool years (3 to 6 years) in both power and reaction to different types of stimuli. Overall, theta band changed from 3.6–5.6 Hz in infants to 4–8 Hz in children and mu band changed from 6.4–8.4 Hz in infants to 8.4–10.4 Hz in children. Also, there were some notable differences and similarities in activation patterns between infants and children. First, infants showed a more widespread theta increase in response to stimulation than preschool children, suggesting reduction and narrowing of task-related cortical activation in development. Second, exploration of unfamiliar objects (attractive toys) caused a predominantly frontal increase of theta power in both infants and children, suggesting control of self-generated goal-related actions. Third, the theta response to social stimulation (social talk in infants and story-telling in children) was strikingly different: theta activity increased predominantly in frontal areas in infants and over parieto-occipital areas in children; this possibly implies stronger control needs in infant to cope with the social interaction and more meaning making and story-related (associative) mental activity in children.

Later in the transition from representational to rule-based though, from 5 to 12 years of age, the relative power of low (delta and theta) vis-a-vis high (alpha and beta) rhythms changed. Somsen et al. (1997) found that power in the delta and theta gradually decreased while power in the alpha and beta changed very little over this age period, implying systematic shift of functioning from sensory-motor-based to control-based rhythms in the transition from representational to rule-based thought. Recently, Rodríguez-Martínez et al. (2015) showed that decreases in theta and beta bands from 6 to 26 years are correlated and they may be related to neuronal pruning. Along this line, Hwang et al. (2012, 2016) explored coupling of beta and alpha bands in the dlPFC and FEF in adolescents and adults asked to inhibit automatic saccade eye movements. They found no age difference in the beta band activity associated with dlPFC functioning, suggesting that this region attains adult levels by adolescence. However, adolescents were weaker in functional inhibition. This was associated with decreased alpha-band power in the FEF and weaker cross-frequency coupling between dlPFC and the FEF, implying limitations in the ability to communicate task-control signals from the dlPFC general control center to the FEF which specializes in the control of eye movements. This pattern might imply that gradually beta oscillations come under the control of low band oscillations but this remains to be directly demonstrated. 

## 4. Individual Differences

The fourth question asked if there are systematic individual differences in brain architecture and functioning that may be connected to individual differences in intellectual functioning and development. Total brain volume is moderately (~0.30) correlated with intelligence (Gignac and Bates 2017). However, the relation is more complicated than this correlation would suggest. For instance, with increasing intelligence or expertice, the brain areas activated to serve a mental task become more refined (Haier 2016). Overall, the various components or material aspects of the brain are differentially related to cognitive functioning. 

Also, individual differences in intelligence are related to brain functioning as such. EEG patterns are highly heritable and so idiosyncratic that they may be used for the fingerprinting of individuals (Buzsáki et al. 2013). There is evidence that highly intelligent (>120 IQ) and less intelligent individuals (<90 IQ) differ in several aspects of brain oscillations. First, more intelligent individuals demonstrate more powerful (higher amplitude) EEG patterns. Second, decreased coherence correlated positively with intelligence. This implies increased spatial differentiation and increased complexity of the brain allowing faster and efficient processing. Third, phase delays, which reflect speed of response of a brain region, are not uniform across the brain. More intelligence individuals show very small phase delays in frontal areas and longer delays in posterior areas. This pattern indicates an efficient frontal command system which swiftly allocates processing to a local integration system that takes on domain specific processing, consuming time and resources (Thatcher et al. 2005). Fourth, there is an optimal balance between phase shift duration and phase lock duration associated with intelligence. Overall, in high intelligence individuals, phase shift (associated with inhibitory activity) is longer and phase lock (associated with excitatory activity) is shorter than in less intelligent individuals. Intelligent individuals give enough time to spot and activate neural assemblies relevant to a task but they are flexible enough to unlock from activated assemblies and shift to new ones. Very fast phase shifting may fail to activate resources and slow shifting may increase noise (Thatcher et al. 2016).

Also, there are extensive changes in development. In the fashion that less intelligent adults activate brain networks more grossly than more intelligence adults, networks are more diffused early in development. Khundrakpam et al. (2017) found that children with higher IQ scores have brain networks that are more highly integrated at both global and local levels. These results further support the idea that individual differences in intelligence are associated with the functional connectivity between parietal and frontal brain regions in both adults and young children, although it is not clear whether this association varies as a function of age.

## 5. Conclusions

We summarized a theory about the architecture and development of the mind and reviewed research on the architecture and development of the brain. This integration is comprehensive and new in that it bridges cognitive developmental theory and research with current research on brain organization, functioning and development. Despite the complexity of the theory, the main idea is simple: brain is an integrated modular system where modules specialize to represent specific aspects of the environment and abstract patterns for each aspect; in humans, there are power abstraction-reduction processes generalizing over abstractions, offering an increasingly highly integrated representation of the environment. These properties are reflected in the organization and development of the developing mind, substantiating psychometric and developmental hierarchical models of cognition and development. Admittedly, this proposed model is vulnerable to criticisms of being weakly grounded on empirical evidence directly related to many of the proposals that are advanced. It is generally recongized that a general weakness of current brain research is that it amassed large volumes of empirical evidence about brain organization, functioning, and change, but this evidence is not integrated into a clear and explicit theoretical framework. This is proposed here, recognizing that the integration may be criticized for weak point-by-point relations between mind entities and brain entities. Below we summarize the main trends and point to the major weaknesses of the endeavor.

### 5.1. Mental and Brain Architecture

The main ternds are as follows. First, the human mind involves (i) several specialized domains of thought and central; (ii) control; (iii) integrative; and (iv) cognizance processes. These mental functions and their interactions are served by several overlapping brain networks. Specifically, (i) several networks rooted in sensory cortices but also extending into various other regions serve the core processes comprising each of the psychological domains. For instance, the anterior temporal lobe may be the semantic hub for the categorical system (Patterson et al. 2007); the angular gyrus in the parietal region may be the hub for the numerical system (Dehaene 2011); the claustrum may serve the visuo-spatial system (Crick and Koch 2005); (ii) Other networks serve control and retention by focusing on and protracting information in time, so that it can be related to preceding or following information. These are rooted in the hippocampus, the reticular formation, and several frontal areas; (iii) Other networks, mainly rooted in temporal, parietal, and prefrontal cortices, take inputs from the networks mentioned above, align them, and abstract their common elements. These networks are associated with reasoning; (iv) Finally, other networks, rooted in frontal and medial cortices, monitor, orient, select, and regulate the networks above to optimize goal-related abstractions. Information entering this last type of networks is available to awareness and metarepresentation. 

Two points need to be emphasized here. First, mental functions are served by overlapping hierarchically organized brain networks rather than regions. Although each network may have a preference for some regions rather than others as their primary entry basis, mental functions are associated with networks rather than regions. Regions are used or reused by multiple networks at multiple spatial scales and they participate in multiple overlapping neural coalitions (Anderson 2014). For instance, entry regions feed associative and abstraction regions with sensory-based content enabling rule- and principle-induction; in turn, these later regions, when activated, call upon the earlier regions to offer exemplary content to substantiate the rules or principles in use, grounding them in the real world (Cao et al. 2019). In fact, there is recent evidence that the same nodes change their network membership according to current cognitive state, suggesting that networks change fluntly according to current cognitive needs (Salehi et al. 2020). Evidently, neural reuse is an important functional aspect of the brain as such. The very process of letting different networks to overlap underlies brain’s capacity to form specific representations of the environment because one component of a network may stand for another component, yielding representational sequences varying in scope and depth. 

In line with this assumption, new methods of analysis suggest that the brain is hierarchically organized from networks in the sensory cortices representing perceptually-based concrete information to networks “deeper” in the brain that are increasingly remote from concrete information, representing abstractions. Network-depth analysis suggests that progressive functional abstraction over network depth may be a fundamental feature of the brain (Taylor et al. 2015). Overall, there seem to be several layers of abstraction in networks. Sensory cortices in the bottom are closer to real inputs and they are more modular. Middle regions, including the hippocampus and MTL, are related to memory and have intermediate distance from sensory inputs. Deep regions, least connected to real inputs, include bilateral inferior temporooccipital, bilateral frontal poles, left cingulate anterior, and left inferior frontal pars triangularis, which may support more abstract cognitive functions, such as reasoning. These networks are activated by practically all sensory or core-specific networks (Yue et al. 2017). Noticeably, symbol creation increases as a function of network depth (and thus abstraction), suggesting that increasing abstraction causes increasing metarepresentation. 

It is important to remember that the early functioning of domain-specific core processes together with their association with specific brain networks has been considered a strong sign that they are innate (Mahon and Caramazza 2011; Carey 2009). The model outlined above suggests that core processes are never free of general processes. Notably, recent research in deep neural networks suggests that the very operation of domain-specific networks may be the result of the operation of a general mechanism in the brain that recognizes and encodes recurrent patterns of information in the environment, related to each of the core processes (Testolin et al. 2020). On top of this, the emergence of mental operations from core processes indicates a general mechanism that supercedes specific core processes, embedding them into more general networks. In evolutionary terms, one might suggest that different modules, such as olfaction, hearing, and vision, evolved as information-specific encoders with inherent generalization functions in sake of their optimal adaptive functioning over very extended time scales (Striedter 2005). Along this line, recent evidence suggests that Broca’s area, traditionally associated with language, involves, additionally to a language network, components associated with domain-general multiple-demand network (Fedorenko and Blank 2020). Thus, modules are generalizers through construction. Additing the one over the other in evolution necessitated a supergeneralizer that would connect and integrate information across these information-specific encoders. These are the higher-level networks noted above. 

It is important to understand the role of consciousness in this processes. There is recent evidence showing that consciousness is sensitive even to processes going on below the threshold of awareness. This system generates a kind of blind insight reflected in the operation of attention and inhibition mechanisms. Blight insight emerges from interactions among predictive processes which yield top-down predictions about the causes of information coming to the senses (e.g., a particular color or face is expected) and bottom-up projections signaling mismatches between expected and observed stimuli (e.g., this was not the color or the face I expected to see). These interactions are registered and metarepresented, allowing systematic self-directed improvement of action in situations where attention is needed (Scott et al. 2014). Tran and Pashler (2017) showed that implicit learning of skills involving fast action, such as tennis learning, involves awareness. Only individuals who explicitly verbalized the rules linking cues to locations learned such predictive rules. Therefore, there may be a cognizance system in the brain (Anderson and Fincham 2014) which “creates, modifies, and rehearses declarative representations of cognitive procedures”. (p. 25), intervening at any of the steps involved in problem solving, thereby creating and inter-relating new representations. Notably, there is evidence that metacognitive confidence evaluations of the accuracy of perceptual discriminations are associated with enhanced functional connectivity as reflected in beta synchrony between motor areas and prefrontal regions (Wokke et al. 2020). This suggests that metacognive awareness integrates sensory evidence and information about ongoing interactions with the world.

### 5.2. Mental and Neuronal Processes

What are the neuronal processes implementing mental processes and mechanisms? It seems that different mental processes associate with different brain rhythms. Entry level processes associated with core operations and specific attention and retention processes are associated with high frequency rhythms (i.e., beta and gamma). Integrative processes, such as reasoning and awareness, are associated with lower frequency rhythms (i.e., delta and theta). These differences also reflect differences in the regions involved (i.e., caudal and central vs. rostral and ventral). 

The brain analogue of AACog lies in the syntactic processes of the brain. Mental comparisons between representations are expressed in oscillatory co-activations between the networks representing the mental entities involved. Ideally, complete rhythm coupling would signify representational alignment. The brain equivalent of abstraction may be the lock of the rhythms coupled above through a rhythm of a different band, such as when several gamma oscillations are bridged by a theta oscillation and the creation of specific markers associated with each abstraction level, standing for commonalities between representations. Cognizance may emerge when this new theta oscillation is made available into a broader theta- or delta-base network, thereby functioning as an autonomous token of a new mental object. Focusing on this new object itself may bring it into the focus of awareness. Ensuing brain activations may be the equivalent of this awareness. The rostrolateral prefrontal cortex is the region primarily serving these needs (Dumontheil 2014). It is important to remember that direct alteration of theta band coordination between frontal regions improves executive control, meaning that theta-based brain activity can directly alter, top-down, brain, and related mental activity based on bands of higher frequencies directly related to perception or action (Jaušovec and Jaušovec 2014; Reinhart 2017).

### 5.3. Mental and Brain Development

How are developmental changes in the brain reflected in developmental changes of cognitive processes? Research suggests that sensory cortices mature early in life, allowing the development of episodic cognition. A control scaffold extends for years in the development of the brain, allowing the rescaling of control at increasingly more inclusive levels. Information maintenance cortices, such as the hippocampus, mature later in infancy and the preschool years, allowing representational encoding of episodic structures. Associative cortices continue to mature well into late childhood and to establish connections with frontal cortices, allowing rule abstraction. 

We focus on the development of the skeletal control network which seems to scaffold development in various functions. The dominant networks are located in the sensory and the motor cortices, the reticular and the parietal, the prefrontal and the frontal cortices, respectively. The crucial aspect of their expansion lies in the addition of extra connections to the parietal and the frontal hubs, integrating and extending the brain network of the previous cycle. We may assume that at each next cycle some further links are added to the left frontoparietal network (lFPN) involving the dlPFC, the IPL, and the rlPFC specified by Vendetti and (Bunge 2014). These links encode further relations in a hierarchical executive or reasoning sequence. Additionally, the cross-coupling of areas may acquire the necessary flexibility in phase shifts and phase locks noted associated by Thatcher et al. (2016) with higher intelligence. This allows shifting between representations of content and representations of relations so that they may optimally be mapped. Finally, this network may be able to activate a substantiation and evaluation phase that calls upon instances (examples or rules) that allow a closing by a decision. At the mental level, these extra additions may be seen in the symbolic units which are episodic in the first cycle (no involvement of the network above), representational but perceptually-dependent in the second cycle (minimal involvement of the network, with probable dominance of the IPL component), rule-based and language encoded in the third cycle (establishment of the network above, with weak involvement of the rlPFC component), and principle-based and language or arbitrary symbol systems (e.g., mathematical) encoded in the fourth cycle (full activation of the network lFPN). Thus, the expansion of the core executive program of each next cycle in representational scope, procedural flexibility, and cognizance resolution parallels brain network expansion, implying that further representational and inferential possibilities become possible because further brain lines are there to support them. 

We view the skeletal executive network as a scaffold for the development of more specialized networks, such as the various logical schemes of deductive reasoning, problem solving strategies in mathematics, moral principles in the social domain, etc. That is, content rich networks are built around the executive scaffold of each phase via a process of translation of the scaffold network into the domain-specific relations and constraints. Building these networks requires the activation of specific circuitry that would carry on the representation of the specific information and relations involved. At both the brain and the mental level, knowledge expansion occurs by adding specific notes peripherally to the main networks. In short, cognitive development involves superimposed sheets of representations in the fashion that brain development involves superimposed sheets of neuronal representations embedded in each other. At the psychological level, higher level representations “are aware” of lower level representations. At the neuronal level, higher level neuronal representations serve as “markers” of lower level representations which may be used to call or activate them, if needed.

We suggested that cognizance rather than speed or working memory is the causal factor for developmental transitions (Demetriou and Spanoudis 2018). The view of consciousness as a recursive system of interactions between a central executive-selection network and other brain systems renders it central in generating new mental content through its continuous rewiring. Specifically, at any time in development, the content and precision of awareness and cognizance depends on the state, differentiation, and synchronization of globally synchronized networks. Thus, in each developmental phase, the awareness possible is commensurate with the network available. We showed that in each next phase increasingly more local networks are hooked onto the global executive network and more long-distance connections are added. This addition goes hand in hand with awareness in each cycle. That is, the resolution and precision of awareness in each cycle reflects the differentiation and tuning of the brain networks involved in consciousness oscillation cycles producing mental units to be availed for attention and executive control. These assumptions may explicate recent strong findings about transfer of cognitive training: transfer may go top-down from relational thought and awareness to attention control and working memory but it does not go bottom-up from these latter processes to fluid intelligence (Christoforides et al. 2016; Papageorgiou et al. 2016; Protzko 2015; Shipstead et al. 2012).

Brain rhythms also change. Overall, with development, activation times drop drastically e.g., the activation time of the ACC, indicated in P300, is 400 msec in children but only 50 msec in adults (Rueda et al. 2005); power increases, but relative power across bands appears to recycle in a fashion reminiscent of mental recycling; cross-band coupling becomes more refined and tuned; networks become more focalized and refined in the fashion that in more intelligent individuals activation is passed on local networks which are precisely modulated by long-distance top-down activation. These changes are reflected in overall changes in processing speed from infancy to adulthoof (Kail 2007). 

However, processing speed and working memory may index cycle and phase transitions in addition to overall development. For instance, when sensorimotor networks are hooked onto a parietal association hub or when, later, this integrated network is hooked onto a pre-frontal hub, sometime is needed to practice and consolidate the new network. Thus, at an initial phase in the functioning of the network, increases in processing speed would reflect changes in the flow of its activation until the core of the network is consolidate. After a certain point in time, the network expands to include already available instances of the lower level networks. At this phase, working memory capacity would be a better psychological marker of network expansion, because it reflects its horizontal expansion and top-down direction in interlinking representations. However, increases in working memory capacity reflect rather than cause increases in the complexity of networks. The findings of Wendelken et al. (2015), indicating that speed was a predictor of change in reasoning at 6–8 years, when dlPFC was dissociated from the vlPFC as a basis of reasoning, and working memory at 8–10 years, when the left and right dlPFC were connected, imply that changes in the rate of change of speed and working memory reflect different phases in the inter-linking between underlying brain networks. These patterns match well with the finding that processing speed is a better index of cognitive changes at the beginning of developmental cycles and working memory at the end (Demetriou et al. 2013, 2014).

### 5.4. Unanswered Questions and Predictions

Obviously, there are many unanswered questions and problems to solve. For example, there is no cognitive function whose corresponding brain structures and networks are fully known. Moreover, we still do not know what is truly general and what is truly specific in both the brain and the mind. Specifically, how much of each general mental function, such as speed, control, or representational capacity, is associated with general brain qualities (i.e., sheer total brain or cortical volume, overall physical state of neurons and neurotransmitters, connectivity, etc.) and how much is accounted for by the fact that particular brain systems (such as the attention or the control networks) are always engaged in cognitive processing? It might be premature to speak about a genetic g because the genes identified to relate with intelligence accounted for a very small amount of variance in intelligence (~5%). However, some genes are associated with various aspects of brain structure and functioning; these, in turn, are associated with individual differences in intelligence and educational attainment. For instance, Sniekers et al. (2017), based on a GWA meta-analysis of 78,308 individuals, identified 15 genomic loci and 40 genes associated with intelligence. These accounted for 4.8% of the variance in intelligence. These genes were associated with synapse formation, axon guidance in brain development, and regulation of myogenic and neuronal differentiation. Hill et al. (2017) found 107 independent associations for intelligence and increased the number of genes involved in intelligence to 338, predicting 7% of individual differences in intelligence. However, intelligence is a polygenic trait, making it premature to speak about a brain g because we still do not know what parts of the brain are common to all mental processes and what parts are specific. Even if g may be associated with the very flexibility in re-wiring and re-linking networks according to changing needs and experience (Barbey 2018), the specific contributions by particular modules of networks at different phases of learning or development is possible. For instance: some aspects of human genetic make-up frame how the brain is formed and functioning; in turn, specific epigenetic events may tune different aspects of the brain to operate high or low, vis-à-vis forthcoming mental challenges. 

Also, we still do not know how each of the various networks carry on its own job (e.g., in terms of rhythms), how the networks interact with each other (e.g., by direct structural connections or by functional coordination), and how they are integrated into a final solution behaviorally and subjectively. In addition, of course, we know very little about how the various types of change in the brain (e.g., myelination, electrical activity, volume, dispersion, activity of neurotransmitters, connectivity, etc.) interact with cognitive developmental changes. 

From the developmental point of view, it is worth examining if recycling in the maturation of brain rhythms reflects recycling in mental development. This would involve the re-working of cross-coupling between low band control rhythms such as delta and theta and alpha and beta bands. One might also assume that spurts in synaptic and cortical pruning may be associated with rewiring and re-alignment of nodal and hub links. This rewiring may coincide with phase and cycle changes at the mental level. It would be interesting to search for band hierarchies in brain synchronization matching cognitive developmental hierarchies where earlier mental achievements are integrated in later achievements. One might hypothesize that very low frequency bands (e.g., delta) control a higher frequency band (e.g., theta), which in turn controls high frequency bands (i.e., alpha, beta, and gamma). Resolving these issues would bring us closer to the grand neuro-cognitive developmental theory of intelligence that would integrate brain with functional maps of mental functions into a common landscape. Obviously, this grand theory is still far ahead of us.

## Figures and Tables

**Table 1:** The three levels of organization of each specialized system of thought.

Domain	Core Processes	Mental Operations	Knowledge and Beliefs
Verbal	Perception according to perceptual similarity; inductive inferences based on similarity-difference relations	Specification of the semantic and logical relations between properties, classification; transformation of properties into mental objects; construction of conceptual systems	Conceptions and misconceptions about the world
Quantitative	Subitization; counting, pointing, bringing in, removing, sharing	Monitoring, reconstruction, execution and control of quantitative transformations, the four arithmetic operations	Factual knowledge about the quantitative aspects of the world, algebraic and statistical inference rules
Spatial	Perception of size, depth, and orientation; formation of mental images	Mental rotation, image integration, image reconstruction, location and direction tracking and reckoning	Stored mental images, mental maps, and scripts about objects, locations, scenes, or layouts maintained in the mind
Causal	Perception of overt and covert causal relations	Trial and error; combinatorial operations; hypothesis formation; systematic experimentation (isolation of variables); model construction	Knowledge, attributions and understanding of the reasons underlying physical and social events and the dynamic aspects of the world
Social	Recognition of conspecifics, recognition of emotionally laden facial expressions	Deciphering the mental and emotional states and intentions of others; organization of actions accordingly; imitation; decentering and taking the other’s perspective	System of social attributions about other persons, their culture, and their society
Linguistic	Use of the grammatical and syntactical structures of language	Identifying truth in information; abstraction of information in goal-relevant ways; differentiation of the contextual from the formal elements; elimination of biases from inferential process; securing validity of inference	Knowledge about grammar, syntax and logical reasoning; metalogical knowledge about nature and justifiability of logical inferences; metacognitive awareness, knowledge, and control of inferential processes
